# Pediatric palliative care for metabolic diseases: 20‐year epidemiological survey of outpatients at a Brazilian quaternary hospital

**DOI:** 10.1002/jmd2.12417

**Published:** 2024-03-31

**Authors:** Gustavo Marquezani Spolador, Clarissa Bueno, Rita Tiziana Verardo Polastrini, Ivete Zoboli, Ana Cristina Henrique, Elaine Freitas, Andréa Gislene do Nascimento, Camila Pugliese, Fernando Kok, Silvia Maria de Macedo Barbosa

**Affiliations:** ^1^ Department of Pain and Pediatric Palliative Care University of Sao Paulo Sao Paulo Brazil; ^2^ Department of Neurometabolic Diseases University of Sao Paulo Sao Paulo Brazil; ^3^ Department of Social Work and Social Care University of Sao Paulo Sao Paulo Brazil; ^4^ Department of Nutrition and Dietetics University of Sao Paulo Sao Paulo Brazil

**Keywords:** inborn errors of metabolism, metabolic diseases, metabolism, palliative care, pediatric palliative care, pediatrics

## Abstract

The interface between pediatric palliative care (PPC) and inborn metabolic diseases (IMD) remains incipient, though these conditions fill the state of art of complex chronic diseases, eligible to this health approach. We analyzed the medical records of PPC clinic during the years 2001 to 2021 and the IMD outpatients. We established a parallel with the world scientific literature concerning the epidemiology of PPC and IMD. Among outpatients, 14% were diagnosed with IMD, which were referred to the PPC service earlier compared to Non‐IMD cases. The Group 3 (complex molecules) was the most frequent (64.7%), following by Group 1 representing by small molecules (21.6%), the latter having a lower median age at diagnosis when compared to the former (0.7 vs. 5.2 years, *p* = 0.001). The sphingolipidoses were the pathologies most frequent in our cohort, in line with what was observed in the literature. There were no differences between IMD groups in terms of diagnosis and PPC referral age, however in Non‐IMD conditions, the age of diagnosis were earlier than IMD. Nevertheless, IMD group showed lower age of referral to PPC. The IMD comprises large fraction of outpatients in the PPC setting, thus further studies are needed in this field.

**Synopsis**

Palliative Care for Inborn Metabolic Diseases: The urgent meeting of two pathways.

## 1 INTRODUCTION

The relationship between pediatric palliative care (PPC) and inborn metabolic diseases (IMD) is still underdeveloped. Despite the availability of disease modifying treatments, most IMD are incurable and require constant monitoring and management of symptoms and dietary interventions.^1^, ^2^

IMD are a heterogeneous group of genetic disorders that affect specific enzymes or biochemical^1^ pathways, leading to impairments in energy production and/or complex molecules metabolism.^3^

Based on their pathophysiological mechanisms, IMD can be categorized into three groups (Box 1): Small molecules or Intermediary Metabolism (Group 1), Energy Metabolism (Group 2), and Complex Molecules (Group 3). This classification facilitates timely clinical diagnosis, complementary testing, and therapeutic interventions.^1^, ^4^

BOX 1 **Pathophysiological classification on inborn metabolic diseases.**

**Table:** 

Groups	Subgroups	Examples	Investigation	Treatment
1 Small molecules	Deficiency	Amino acids synthesis, neurotransmitters, metal	Amino acids (P, U, CSF), monoamines, neopterin, metal	Some untreatable, amino acid replacement, metal supplementation
	Accumulation	Amino acids catabolism, organic Acidurias, vitamin B, Urea Cycle Disorders	Amino acids (P, U, CSF), organic acids (U), acylcarnitines (P, U)	Toxin removal, special diets, scavenger drugs, extra‐corporeal procedures, vitamins
2 Energy metabolism	Carriers	GLUT1, GLUT2, MCT1, MCT8	Glucose (CSF), thyroid profile, creatine (MRI)	Special diets, creatine, T3 analogues
	Cytoplasm	Glycogen Storage Disease, insulin defects, creatine defects	Glucose (P), lactate, ketone bodies, uric acid, creatine (P, U), insulin, creatine (MRI)	Special diets, creatine, glucagon
	Mitochondrial	Fatty acid oxidation, ketones metabolism, Krebs Cycle, Pyruvate/lactate oxidation	Functional tests, lactate, pyruvate, amino acids, organic acids, acylcarnitines, molecular tests	Special diets, riboflavin, carnitine
3 Complex molecules	Deficiency	Glycogen Storage Disease, SLC10A7, MFSD2A, sphingolipids, cholesterol and bile acids, peroxisomal	VLCFA (F), molecular test	BMT, Untreatable
	Accumulation	Glycogen Storage Disease, MPS, Niemann‐Pick Sialidosis	Enzymatic assay, VLCFA (F), molecular test	Special diets, live transplantation, ERT, BMT
	Traffic and Processing	CDG, autophagy, synaptic vesicle	TflEF, molecular test	Untreatable

Abbreviations: BMT, bone marrow transplantation; CDG, congenital disorder of glycosylation; CSF, cerebrospinal fluid; ERT, enzyme replacement therapy; F, fibroblast; MPS, mucopolysaccharidosis; MRI, magnetic resonance imaging; P, plasma; TfIEF, transferrin isoelectric focusing; U, urine; VLCFA, very long chain fatty acids.

Once IMD has affected multiple systems or organs to the extent that they necessitate specialized medical follow‐up in a tertiary center, they qualify as complex chronic diseases (CCC).^5^

Hence, given the high demands on health services, such as hospitalizations, intensive care units, surgical procedures, and multidisciplinary teams, that characterize the course of IMD, PPC assumes a vital role in the well‐being of these patients and their families.^6^, ^7^

The World Health Organization (WHO) defines PPC as holistic care that addresses the mind, spirit, and body of the patient, as well as the family support, starting from the diagnosis of a CCC and continuing throughout the life span along with the disease‐modifying treatment. In line with this definition, the WHO also identified five groups of diseases that are eligible for PPC, based on the concept of CCC and presented in Table 1.^2^

**TABLE 1:** Complex chronic diseases eligible to pediatric palliative care according WHO.

Group	Description	Examples
Group 1	Conditions for which curative treatment is possible but may fail	Cancer and congenital heart diseases
Group 2	Conditions that require complex and prolonged treatment	Cystic fibrosis, epidermolysis, bullosa, and neuromuscular diseases
Group 3	Life‐limiting progressive conditions without curative treatment	Inborn errors of metabolism and chromosomal disorders
Group 4	Non‐progressive, irreversible conditions that cause major impairment leading to health complications and premature death	Cerebral palsy, extreme prematurity, and central nervous system malformations
Group 5	Unborn children with major health problems, children who survive for a few hours or days, children with congenital anomalies with endangered vital function	Congenital heart disease and skeletal dysplasias

*Source*: WHO (2018).

Based on these five groups, we can adjust them to the scenario of IMD by considering some disorders of amino acid catabolism, organic aciduria, and urea cycle defects as candidates for organ transplantation, which may have a curative effect, but also a risk of failure, as exemplified in Group 1.

Moreover, besides the aforementioned conditions, fatty acid oxidation disorders and glucose transporter defects demand complex and prolonged treatment, meeting the criteria of Group 2.

And in accordance with the notion of Group 4, amino acid synthesis defects, metabolic repair defects, trafficking and processing disorders, and mitochondriopathies can be classified as such.

Nonetheless, there is scarce literature on the chronic assessment of patients with inborn errors of metabolism in the palliative care context, which warrants the execution of this article.

## 2 MATERIALS AND METHODS

### 2.1 Patients and databases

This study is a joint effort of the PPC and neurometabolic units of a quaternary hospital in Sao Paulo, Brazil.

The inclusion criteria consisted of: (1) confirmed or suspected diagnosis of IMD; (2) at least 1 year of PPC outpatient monitoring; (3) concurrent follow‐up with the neurometabolic team.

### 2.2 Epidemiology of IMD in PPC setting

We conducted a comparative analysis of the main diagnosis of IMD at the PPC clinic and the global literature. We then established a correlation between the most prevalent pathophysiological groups and the age of diagnosis, referral, and length of referral to PPC.

### 2.3 Clinical data

This is a retrospective study that obtained the clinical data on the initiation of the PPC surveillance from the medical records of a quaternary hospital in Sao Paulo, Brazil, between 2001 and 2021.

### 2.4 Statistical analysis

To analyze the data, we followed these steps: First, the assumptions of normality and homogeneity of variance were checked using the Shapiro Wilk and the Levene tests, respectively. These tests are important to ensure the validity of the parametric tests used later. Second, the data were presented using absolute and relative frequencies (%) for qualitative variables and median and interquartile range for quantitative variables. Third, the two groups of patients were compared: those with IMD and those without (Non‐IMD). We used the Chi‐square test (2 × 2) for qualitative variables and the Mann–Whitney *U* test for quantitative variables. These tests are appropriate for comparing two independent groups with non‐normal data.

Finally, we compared the three subgroups of IMD patients based on their pathophysiological mechanisms: small molecules or intermediary metabolism (Group 1), energy metabolism (Group 2), and complex molecules (Group 3). We used the Chi‐square test for qualitative variables and the Kruskal–Wallis test followed by the Mann–Whitney *U* test with Bonferroni correction for pairwise comparisons for quantitative variables. These tests are suitable for comparing more than two independent groups with non‐normal data. All analyses were performed using the PASW statistics 25.0 software (SPSS Inc., Chicago, USA), a widely used program for statistical analysis in social sciences. A significance level (*α*) of 5% (*p* < 0.05) was adopted.

## 3 RESULTS

From 2001 to 2021, we referred and followed up 363 patients at the PPC clinic. Of these, 51 patients (14.0%) had IMD (Table 2). The gender distribution of the IMD patients was 20 females (39.2%) and 31 males (60.8%), which did not differ significantly from the Non‐IMD group (*p* = 0.275). The IMD patients had a higher median age of diagnosis than the Non‐IMD patients (5.0 vs. 0.0 years, *p* < 0.001), but a lower median age of PPC referral (5.8 vs. 9.5 years, *p* = 0.001).

**TABLE 2:** Outpatients conditions followed up by pediatric palliative care (*n* = 363).

	IMD (*n* = 51)	Non‐IMD (*n* = 312)	*p*‐Value
Gender, *n* (%)			
Female	20 (39.2%)	148 (47.4%)	0.275^CS^
Male	31 (60.8%)	164 (52.6%)	
Age of diagnosis (years), median (interquartile range)	5.0 (0.9–7.9)	0.0 (0.0–0.075)*	<0.001^MW^
Ages of PPC referral (years), (interquartile range)	5.8 (1.2–11.1)	9.5 (5.0–14.4)*	0.001^MW^

Abbreviation: IMD, inherited metabolic diseases.

* Significant difference from the IMD group. CS: *p*‐value for Chi‐square test; MW: *p*‐value for Mann–Whitney *U* test.

As shown in Table 3, the majority of IMD patients belonged to Group 3 (64.7%, *p* < 0.001), which comprised complex molecule disorders. Patients in Group 2, which included energy metabolism disorders, had a significantly lower median age of diagnosis than those in Group 3 (0.7 vs. 5.2 years, *p* = 0.001). However, the difference between Group 2 and Group 1, which consisted of small molecule or intermediary metabolism disorders, was not statistically significant (0.7 vs. 5.8 years, *p* = 0.094). This may be attributed to the small sample size of these two groups. No significant differences were found among the groups regarding the age of PPC referral (*p* = 0.437) and the duration from diagnosis to PPC referral (*p* = 0.873).

**TABLE 3:** Aspects of IMD patients into pediatric palliative care (*n* = 51).

	Group 1	Group 2	Group 3	*p*‐Value
Number of patients, *n* (%)	11 (21.6%)	7 (13.7%)	33 (64.7%)	<0.001^CS^
Age of diagnosis (years), median (interquartile range)	5.8 (0.2–10.5)	0.7 (0.5–1.4)*	5.2 (2.5–8.9)	0.012^KW^
Age of PPC referral (years), median (interquartile range)	11.7 (7.6–14.5)	2.6 (1.7–15.2)	9.5 (5.5–14.2)	0.437^KW^
Time between diagnosis and PPC referral (years), median (interquartile range)	2.6 (1.1–6.2)	1.8 (1.0–14.3)	2.8 (1.2–4.5)	0.873^KW^

* Significant difference from Group 3; CS: *p*‐value for Chi‐square test; KW: *p*‐value for Kruskal–Wallis test; Group 1 (small molecules or intermediary metabolism); Group 2 (complex molecules); Group 3 (energy metabolism).

## 4 DISCUSSION

This study investigated the epidemiological characteristics of IMD outpatients who received PPC, compared them with Non‐IMD patients, and examined the timing of referral to this service. This is the first study in Brazil that explores the similarities and differences between these two groups of patients.

In the field of epidemiology, particularly in relation to IMD, a critical factor is the coefficient of inbreeding. This coefficient represents the probability that two alleles at a specific locus are identical by descent.^8^ The values of this coefficient vary significantly across different regions. For instance, in Canada, it ranges from 0.00004 to 0.0008, while in Saudi Arabia, it is considerably higher at 0.024.^9^ In contrast, Brazil exhibits a mean coefficient of inbreeding of 0.0023.^10^

The proportion of PPC patients with IMD was 14%, which is consistent with the few existing studies that report frequencies between 14.6%^6^ and 19.7%.^11^ The IMD group had a higher median age of diagnosis than the Non‐IMD group, which can be attributed to the composition of the palliative care cohort, which included epidermolysis bullosa (36.9%) and non‐IMD neurological diseases (19.0%). These conditions have easier clinical diagnosis and require less specialized tests, unlike IMD, which are limited by the availability of newborn screening tests in Brazil. The IMD group also had an earlier median age of referral to PPC, possibly because they had multisystem pathologies that demanded more symptom management and greater awareness of the main team regarding the concept of PPC.

There were no significant differences between the three IMD groups in terms of the age of referral to PPC and the time interval between diagnosis and referral. However, when compared with another similar study, the median age of referral to PPC in our study was later.^11^

We observed a higher frequency of Group 3 conditions, such as sphingolipidoses (13 patients), adrenoleukodystrophy (9 patients), mucopolysaccharidosis (7 patients), and neuronal ceroid lipofuscinoses (4 patients). This prevalence was also reported in the literature among inpatients and outpatients, with the most common diseases being sphingolipidoses (10 patients) and mucopolysaccharidosis (2 patients).^8^, ^11^ The Group 1 conditions were the second most frequent in the PPC setting and comprised neurodegeneration with brain iron accumulation (NBIA) (6 patients), organic acidurias (4 patients), and Lesch–Nyhan syndrome (1 patient). In contrast, in other studies, this group was the least frequent among inpatients (4 patients) and outpatients (4 patients), mainly presenting amino acid metabolism disorders.^8^, ^11^ The Group 2 conditions were the least frequent in our study, and we found a discrepancy with the other studies, which mainly identified mitochondrial disorders, while our population also had beta oxidation and vitamin disorders.^8^, ^11^

## 5 CONCLUSION

Given that patients with IMD constitute approximately 14% of the PPC population, there is a paucity of literature that chronicles the long‐term evaluation of these patients within the palliative care framework. This is particularly true when it comes to delineating the epidemiology of the most prevalent pathologies and drawing parallels between the age of diagnosis and the age of referral. Therefore, there is a pressing need for more multicentric studies that can provide a more comprehensive epidemiological and symptomatological profile, particularly in the context of the specific country where the studies are conducted. This will contribute significantly to the body of knowledge in this field and potentially lead to improved patient care strategies.

## AUTHOR CONTRIBUTIONS

**Gustavo Marquezani Spolador**: Conceptualization; formal analysis; investigation; resources; writing – original draft. **Clarissa Bueno**: Formal analysis; investigation; resources. **Rita Tiziana Verardo Polastrini**: Formal analysis; investigation; resources. **Ivete Zoboli**: Visualization; supervision. **Ana Cristina Henrique**: Visualization. **Elaine Freitas**: Visualization. **Andréa Gislene do Nascimento**: Visualization. **Camila Pugliese**: Visualization. **Fernando Kok**: Supervision; project administration. **Silvia Maria de Macedo Barbosa**: Supervision; project administration.

## FUNDING INFORMATION

This work was supported by author's resources.

## CONFLICT OF INTEREST STATEMENT

The authors declare no conflicts of interest.

## ETHICS STATEMENT

The study protocol underwent rigorous scrutiny and was subsequently approved by the Ethics Committee of the Universidade de São Paulo (USP), bearing the approval number CAAE 69446222.1.0000.0068. In accordance with the ethical guidelines stipulated by the same institution, written informed consent was procured from a majority of the families involved in the study. It should be noted that some patients were lost to follow‐up, thereby precluding the possibility of obtaining their consent. Despite this, all procedures were conducted in strict adherence to the ethical standards set forth by the aforementioned Ethics Committee.

## Data Availability

Clinical data were extracted from the electronic medical records maintained by the Pediatric Palliative Care and Pediatric Neurology Outpatient Clinics of the Child Institute of Clinic Hospital (HC‐FMUSP). With respect to the epidemiology of inborn errors of metabolism, these conditions will be systematically classified into pathophysiological groups, namely intoxication, energy, and complex molecules. This categorization will facilitate a more nuanced understanding of the prevalence and impact of these metabolic disorders.

## References

[1] Saudubray JM , Mochel F , Lamari F , Garcia‐Cazorla A . Proposal for a simplified classification of IMD based on a pathophysiological approach: a practical guide for clinicians. J Inherit Metab Dis. 2019;42(4):706‐727. 30883825 10.1002/jimd.12086

[2] Integrating Palliative Care and Symptom Relief into Paediatrics: A WHO Guide for Health Care Planners. World Health Organization; 2018.

[3] Morava E , Rahman S , Peters V , Baumgartner MR , Patterson M , Zschocke J . Quo vadis: the re‐definition of “inborn metabolic diseases”. J Inherit Metab Dis. 2015;38(6):1003‐1006. 26420281 10.1007/s10545-015-9893-x

[4] Jones P , Patel K , Rakheja D . Introduction. In: Jones P , Patel K , Rakheja D , eds. A Quick Guide to Metabolic Disease Testing Interpretation. 2nd ed. Academic Press; 2020:1‐14.

[5] Feudtner C , Hexem K , Rourke MT . Epidemiology and the care of children with complex conditions. In: Wolfe J , Hinds P , Sourkes P , eds. Textbook of Interdisciplinary Pediatric Palliative Care. Elsevier; 2011:7‐8.

[6] Schwantes S , O'Brien HW . Pediatric palliative care for children with complex chronic medical conditions. Pediatr Clin North Am. 2014;61(4):797‐821. 25084725 10.1016/j.pcl.2014.04.011

[7] Hoell JI , Warfsmann J , Distelmaier F , Borkhardt A , Janßen G , Kuhlen M . Challenges of palliative care in children with inborn metabolic diseases. Orphanet J Rare Dis. 2018;13(1):112. 29986738 10.1186/s13023-018-0868-5 PMC6038293

[8] Rousset F . Inbreeding and relatedness coefficients: what do they measure? Heredity (Edinb). 2002;88(5):371‐380. 11986874 10.1038/sj.hdy.6800065

[9] Moammar H , Cheriyan G , Mathew R , Al‐Sannaa N . Incidence and patterns of inborn errors of metabolism in the Eastern Province of Saudi Arabia, 1983‐2008. Ann Saudi Med. 2010;30(4):271‐277. 20622343 10.4103/0256-4947.65254 PMC2931777

[10] Santos S , Kok F , Weller M , de Paiva FR , Otto PA . Inbreeding levels in Northeast Brazil: strategies for the prospecting of new genetic disorders. Genet Mol Biol. 2010;33(2):220‐223. 21637472 10.1590/S1415-47572010005000020 PMC3036858

[11] Harputluoğlu N , Köse M , Yılmaz Ü , Çelik T . Inborn errors of metabolism in palliative care. Pediatr Int. 2021;63(10):1175‐1179. 33600034 10.1111/ped.14660

